# Explainable Connectionist-Temporal-Classification-Based Scene Text Recognition

**DOI:** 10.3390/jimaging9110248

**Published:** 2023-11-15

**Authors:** Rina Buoy, Masakazu Iwamura, Sovila Srun, Koichi Kise

**Affiliations:** 1 Department of Core Informatics, Graduate School of Informatics, Osaka Metropolitan University, Osaka 599-8531, Japan; 2 Department of Information Technology Engineering, Faculty of Engineering, Royal University of Phnom Penh, Phnom Penh 12156, Cambodia

**Keywords:** vision Transformer, connectionist temporal classification, scene text recognition, character localization, model explainability

## Abstract

Connectionist temporal classification (CTC) is a favored decoder in scene text recognition (STR) for its simplicity and efficiency. However, most CTC-based methods utilize one-dimensional (1D) vector sequences, usually derived from a recurrent neural network (RNN) encoder. This results in the absence of explainable 2D spatial relationship between the predicted characters and corresponding image regions, essential for model explainability. On the other hand, 2D attention-based methods enhance recognition accuracy and offer character location information via cross-attention mechanisms, linking predictions to image regions. However, these methods are more computationally intensive, compared with the 1D CTC-based methods. To achieve both low latency and model explainability via character localization using a 1D CTC decoder, we propose a marginalization-based method that processes 2D feature maps and predicts a sequence of 2D joint probability distributions over the height and class dimensions. Based on the proposed method, we newly introduce an association map that aids in character localization and model prediction explanation. This map parallels the role of a cross-attention map, as seen in computationally-intensive attention-based architectures. With the proposed method, we consider a ViT-CTC STR architecture that uses a 1D CTC decoder and a pretrained vision Transformer (ViT) as a 2D feature extractor. Our ViT-CTC models were trained on synthetic data and fine-tuned on real labeled sets. These models outperform the recent state-of-the-art (SOTA) CTC-based methods on benchmarks in terms of recognition accuracy. Compared with the baseline Transformer-decoder-based models, our ViT-CTC models offer a speed boost up to 12 times regardless of the backbone, with a maximum 3.1% reduction in total word recognition accuracy. In addition, both qualitative and quantitative assessments of character locations estimated from the association map align closely with those from the cross-attention map and ground-truth character-level bounding boxes.

## 1. Introduction

Scene text recognition (STR) identifies text in natural scenes and remains a vibrant research field due to challenging imaging conditions [1,2]. Current deep learning methods for STR typically comprise a visual feature extractor, a sequence modeler, and a decoder. The choice of decoder significantly impacts model recognition performance, latency, and explainability, given the same feature extractor and sequence modeler design. State-of-the-art (SOTA) methods categorize by their decoding of visual features into characters using connectionist temporal classification (CTC) and attention-based and Transformer decoders [3,4,5].

**Figure 1:**
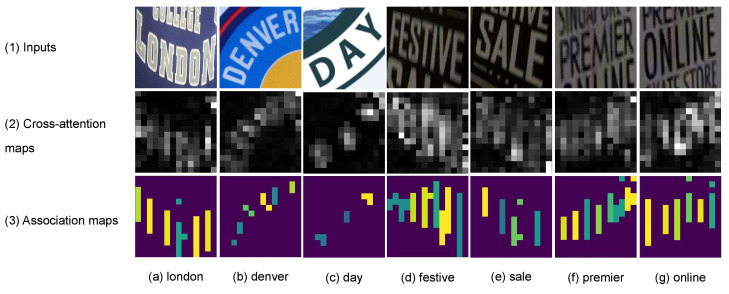
The cross-attention vs. the association maps. The first row consists of text images. The second and third rows consist of the cross-attention and association maps, respectively, that associate each predicted character with image regions. The last row consists of text transcriptions. The cross-attention map is obtained from a Transformer decoder, while the association map is obtained from a ViT-CTC model. Best viewed in color.

A 2D attention-based or Transformer decoder, using 2D feature maps, excels in recognition accuracy and character localization through a cross-attention mechanism. Unlike Transformer-based object detectors, such as DETR [6] and V-DETR [7], which directly output object bounding boxes, a Transformer-based text recognizer outputs only characters. These characters can be localized via the decoder’s cross-attention map. With enough inductive biases, including locality, the Transformer decoder attends only to the locations of the objects of interest [7]. Thus, the Transformer decoder generates a cross-attention map, linking predicted characters to relevant image regions. This location information yields benefits like model explainability [8,9,10,11,12] and text rectification [13]. Figure 1(2) exemplifies the overlaid cross-attention maps (summed across predicted characters) from a Transformer decoder, illustrating alignment between character positions and attention weights. However, it should be noted that the attention-based decoder has high latency due to an intricate attention mechanism [3,14].

Conversely, the CTC decoder offers superior latency efficiency but often sacrifices recognition accuracy compared with the attention-based decoder [3,4,14]. The CTC decoder demands a 1D class probability distribution sequence input, prompting the common use of a 1D feature extractor in existing CTC-based methods [14,15,16,17,18]. However, this approach hampers the ability to establish explainable 2D spatial relationship between the predicted characters and relevant image regions. The 2D-CTC [19] method emerged to handle 2D feature maps, extending the 1D CTC algorithm to process the height dimension. However, using 2D-CTC involves a trade-off, resulting in higher inference latency and training costs, particularly with larger 2D feature maps.

For explainable character localization using a 1D CTC decoder, we introduce a ViT-CTC STR architecture that enables a 1D CTC decoder with a pretrained vision Transformer (ViT) to act as a 2D feature extractor. To incorporate the 2D feature extractor, we propose a novel marginalization-based technique that predicts 2D joint probability distributions over the height and class dimensions. By marginalizing the height dimension, we obtain a 1D class probability distribution sequence suited for a 1D CTC decoder.

Our proposed method also generates an association map, serving for character localization and model prediction explanation. This map resembles the role of a cross-attention map in the attention-based architectures but with significantly lower computational demand. Qualitative comparisons between the overlaid cross-attention and association maps are depicted in Figure 1(2),(3), respectively, showcasing alignment. Moreover, unlike 2D-CTC [19], our method maintains consistent inference latency and training cost, regardless of 2D feature map size. To quantitatively measure the alignment between character positions from the association map and the ground-truth character locations, we propose an alignment evaluation metric (AEM).

Our contributions can be summarized as follows:

1. We introduce a novel marginalization-based method for enabling a 2D feature extractor to be compatible with a 1D CTC decoder. This method yields an association map that links predicted characters to relevant image regions, enabling character localization and improving prediction explainability.
2. We derive an alignment evaluation metric (AEM) that measures the alignment between character positions from the association map and the ground-truth character locations. This metric can also be used for the cross-attention map.
3. Using our method, we experimented with the ViT-CTC architecture with various pretrained ViT backbones and a 1D CTC decoder. Our ViT-CTC models outperform the recent SOTA methods on public benchmark datasets.
4. Compared with a Transformer-decoder-based model, a ViT-CTC model offers a remarkable speed boost, surpassing the former by up to 12 times, regardless of the ViT backbone used. This speed gain comes with a maximum reduction in total word recognition accuracy of 3.1%. Hence, the ViT-CTC model is particularly attractive for low-latency, resource-constrained environments.

### 1.1. Related Work

In this section, we provide a brief review of common decoders in mainstream scene text recognition (STR) architectures. In addition, we also describe the recent advances of vision Transformer (ViT) architectures and their adoptions in STR, followed by model explanation through visualizations.

#### 1.1.1. Scene Text Recognition

Scene text recognition is a variant of unsegmented sequence labeling tasks in which a 2D input stream of pixels is labeled with a sequence of characters. Other similar perceptual tasks include speech and gesture recognition [20].

Graves et al. [20] introduced the CTC algorithm, which maps a recurrent neural network (RNN) output sequence of a speech signal to a character sequence. CTC incorporates a blank token (ϵ) to handle multiple input-to-output alignments. Instead of predicting a probability of a single alignment, CTC estimates a total probability by marginalizing over all possible alignments.

CTC gained popularity in text recognition, leading to numerous CTC-based STR methods [14,15,16,17,18]. These methods typically employ a common pipeline encompassing optional rectification, a 1D convolutional feature extractor, a recurrent sequence modeler, and a 1D CTC decoder. While most CTC-based methods were initially designed for the Latin script, Gunna et al. [21] and Hu et al. [4] extended the CTC-based recognition pipeline to different Indian and Vietnamese scripts, respectively. However, a 1D CTC-based approach (using a 1D feature extractor) is unable to establish explainable 2D spatial relationships between predicted characters and relevant image areas.

To tackle this, 2D-CTC [19], an extension of the 1D CTC algorithm with the height dimension, handles 2D feature maps. However, it leads to increased inference latency and training cost, particularly based on the height of feature maps. Moreover, there is a lack of standardized, optimized 2D-CTC implementations in prevalent deep learning frameworks.

In contrast to a 1D CTC decoder, an attention-based decoder accommodates both 1D and 2D feature extractors. One-dimensional attention-based methods [4,14,22,23] substitute a CTC decoder with an attention-based one to enhance recognition performance by capturing character dependencies. Recognizing limitations in accurately predicting characters within complex and curved text, 2D attention-based methods [9,24] emerged.

As Transformer networks [10] gained prominence, the Transformer decoder became the standard attention-based decoder, leading to Transformer-decoder-based methods [25,26]. Via cross-attention mechanisms, the attention-based decoder produces a cross-attention map, associating each predicted character with relevant input image regions. The cross-attention map is widely used for visual explanations of model predictions [8,9,10,11,12]. Despite its superior performance, Baek et al. [14] showed that an attention-based decoder, using the same feature extractor, yields about three times higher latency than a CTC-based decoder.

#### 1.1.2. Vision Transformer

Transformers [10] have established themselves in natural language processing (NLP). Vision Transformers (ViT) [27] extend this architecture to vision tasks by dividing images into patches and projecting them as tokens, similar to words in NLP. The ViT’s training demands are computationally efficient, but it lacks inductive biases. Addressing this, effective ViT models require substantial training data (priors). Data-efficient image Transformers [28,29,30] were introduced to alleviate data demands, achieving competitive outcomes against convolutional networks. ViT swiftly integrated into existing STR setups as a 2D feature extractor and sequence modeler. ViT-based STR methods [1,5,31] were subsequently proposed, displaying SOTA performance, particularly when trained on real labeled data.

#### 1.1.3. Visual Model Explanations

To help users understand model failure and discover biases in training data, transparent models are necessary [32]. Nevertheless, deep neural networks (DNNs) behave as black boxes, making them difficult to understand. According to Junkang and Joe [32], an explanation map is a map that highlights relevant regions that contribute to a model’s decision. The explanation can be obtained by using class activation mapping (CAM)-based or attention-based methods. Gradient-weighted class activation mapping (Grad-CAM) [33] is an example of CAM-based methods. Grad-CAM computes the gradients of a given class to produce a low-resolution localization map that highlights relevant image regions. Xu et al. [8] utilized an attention mechanism and visualized the attention map to show human intuition-like alignments between a model-generated caption and relevant image regions.

## 2. Materials and Methods

### 2.1. Proposed Method

In our study, ViT-CTC models leverage pretrained vision Transformers and a 1D CTC decoder. This allows our models to draw on extensive visual pretraining and exploit 2D spatial feature relationships via self-attention layers, all while retaining the low latency of a 1D CTC decoder. The introduced marginalization-based method also facilitates character localization and model prediction explanations through a novel association map that is absent in the existing 1D CTC-based methods.

In this section, we present the details of our proposed marginalization-based method in 2D class probability space. We begin by providing a concise overview of the 1D CTC algorithm and its assumptions in Section 2.1.1, followed by the detailed derivations of the proposed method in Section 2.1.2. We formulate the association map that relates each model prediction to relevant image regions in Section 2.1.3. Lastly, we derive an alignment evaluation metric (AEM) that measures the alignment between character locations estimated using the association and cross-attention maps and ground-truth character locations in Section 2.1.4.

#### 2.1.1. Connectionist Temporal Classification (CTC)

CTC assigns a total probability of an output sequence (Y) given an input sequence (X) [20,34,35]. Instead of assigning a probability to the most likely alignment, CTC estimates a total probability by summing over all possible alignments between an input and output sequence. CTC introduces a blank or no-label token (ϵ) to allow the alignments and the input to have the same length. For any alignment, repeated characters are merged and blank tokens are removed to produce a final output sequence. For example, $A_{1}=\left(\epsilon ,c,\epsilon ,a,\epsilon ,t\right)$ and $A_{2}=\left(c,c,\epsilon ,a,\epsilon ,t\right)$ are two of the possible and valid alignments for the same word, *cat*. Mathematically, the total probability assigned by CTC is given by [20,34,35]

(1) $$p\left(Y|X\right)=\sum_{A\in S_{X,Y}}\prod_{t=1}^{W^{\prime }}p_{t}\left(a_{t}|X\right),$$

where p(Y|X) is a total probability of an (X,Y) pair. $A=(a_{1},\ldots ,a_{W^{\prime }})$ is an alignment and $S_{X,Y}=\left(A_{1},\ldots ,A_{n}\right)$ is a set of possible and valid alignments between X and Y. $p_{t}\left(a_{t}|X\right)$ is a conditional probability on X at a prediction frame, *t*. Thus, at each timestep *t*, a learning algorithm must produce a valid probability distribution (i.e., 1D vector) over characters. In the context of text recognition, the width dimension is treated as time while the height dimension is often collapsed by convolution and pooling layers.

Since $S_{X,Y}$ can be large, naive implementation is computationally inefficient. This is mitigated by dynamic programming by merging two alignments with the same output at the same *t*. Modern deep learning libraries have a built-in, optimized, efficient, low-level implementation of CTC. During inference, a greedy decoding scheme is used by selecting the most likely output at each prediction frame independently to obtain the highest probability alignment, $A^{*}$, from which ϵ and duplicate characters are removed and merged, respectively [34]. The greedy and parallel decoding nature allows CTC to achieve low latency that is crucial in low-resource and real-time environments. $A^{*}$ is given by

(2) $$A^{*}=\underset{A}{\mathrm{argmax}}\prod_{i=1}^{T}p_{t}\left(a_{t}|X\right).$$

The CTC algorithm makes the following assumptions [34]:

1. Conditional independence. The predicted characters are conditionally independent, meaning there are no dependencies between characters.
2. Monotonicity. When handling the subsequent feature vector, the current character can persist or the subsequent character must be processed.
3. Many to one. There can be multiple feature vectors corresponding to a single output character. This implies that the length of feature vectors must be greater than or equal to the length of target characters.

#### 2.1.2. The Proposed Marginalization-Based Method

The concept of the proposed method is to handle 2D feature maps with a 1D CTC decoder without adding complexity. This is achieved by applying the marginalization rule in 2D class probability space.

**Figure 2:**
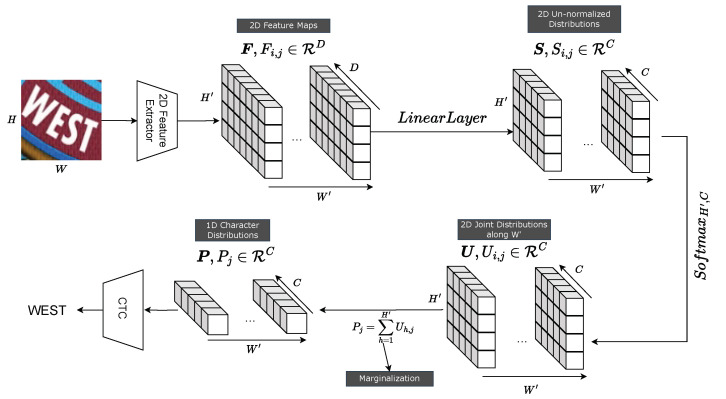
The proposed marginalization-based method: A 2D feature sequence, $F=(F_{1,1},\ldots ,F_{H^{\prime },W^{\prime }})$, is produced by a 2D feature extractor such as a ViT backbone. F is fed to a linear layer to produce $S=(S_{1,1},\ldots ,S_{H^{\prime },W^{\prime }})$ from which a softmax normalization is performed over both $H^{\prime }$ and *C* dimensions. Next, the normalized $U=(U_{1,1},\ldots ,U_{H^{\prime },W^{\prime }})$ is marginalized over the $H^{\prime }$ dimension to produce $P=(P_{1},\ldots ,P_{W^{\prime }})$ that is fed to a CTC decoder. *D* and *C* are the feature and class dimensions, respectively.

Concretely, as shown in Figure 2, a ViT encoder takes an input image and produces 2D feature maps, represented by $F=(F_{1,1},\ldots ,F_{H^{\prime },W^{\prime }})$, $F_{i,j}$∈$R^{D}$, where $H^{\prime }$, $W^{\prime }$, and *D* are the height, width, and embedding dimensions of the feature maps. F is directly fed to a linear layer to produce unnormalized 2D score distributions, $S=(S_{1,1},\ldots ,S_{H^{\prime },W^{\prime }})$, $S_{i,j}$∈$R^{C}$. S is given by

**Figure 3:**
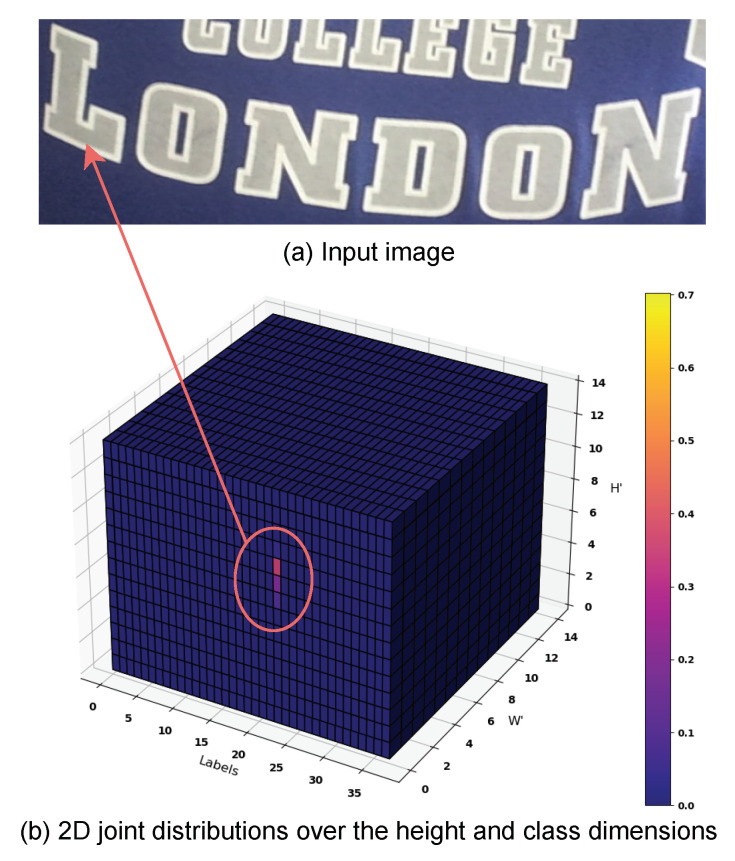
3D graphical illustration of U for an input image. (**a**) Input image. (**b**) The computed U. At $W^{\prime }=1$, the bright cells, responding to the character ***L***, have a high probability. Best viewed in color.

(3) S=LinearLayer(F),

where **LinearLayer** is a feedforward neural network. Each $S_{i,j}$ is an unnormalized vector and C is the number of class labels. A softmax normalization is applied to S along both $H^{\prime }$ and *C* dimensions to produce $U=(U_{1,1},\ldots ,U_{H^{\prime },W^{\prime }})$, $U_{i,j}$∈$R^{C}$. U is given by

(4) $$U={\mathrm{Softmax}}_{H^{\prime },C}\left(S\right),$$

where ${\mathrm{Softmax}}_{H^{\prime },C}$ is a softmax operator along the $H^{\prime }$ and *C* dimensions. A cross-section along $W^{\prime }$ is a valid 2D joint probability distribution over the $H^{\prime }$ and *C* dimensions. A 3D graphical illustration of U is provided in Figure 3.

Next, U is marginalized over the $H^{\prime }$ dimension to produce a sequence of valid 1D probability distributions over the C dimension, $P=(P_{1},\ldots ,P_{W^{\prime }})$, $P_{j}$∈$R^{C}$, that is required by a CTC decoder. $P_{j}$ is given by

(5) $$P_{j}=\sum_{h=1}^{H^{\prime }}U_{h,j},$$

where each $P_{j}$ is a normalized class probability distribution vector. In the case of a 1D feature extractor (i.e., $H^{\prime }=1$), U is exactly P. The overall text recognition workflow with the proposed method is shown in Figure 2.

Beyond the CTC algorithm’s assumptions, our proposed method assumes horizontal or curved textline, excluding vertical orientation.

#### 2.1.3. Association Map (AM)

In the existing CTC-based methods, the height dimension is physically discarded by feature averaging or pooling layers. The proposed method preserves the height dimension, making a 2D feature extractor compatible with a CTC decoder.

Thanks to the proposed method, a cross-section along the $W^{\prime }$ dimension of U forms a valid 2D joint probability distribution over the $H^{\prime }$ and *C* dimensions, as shown in Figure 3. Based on U, we can derive a novel association map (AM) that enables linking each predicted character to relevant image regions. This spatial connection serves two purposes: (1) explaining model predictions and (2) character localization.

The association map functions in the same way as the localization map of Grad-CAM [33], but without gradients, and the attention map [8], but without an attention mechanism. Concretely, given the most likely alignment, $A^{*}=\left(a_{1},\ldots ,a_{W^{\prime }}\right)$, $\mathrm{AM}=({\mathrm{AM}}_{1,1},\ldots ,{\mathrm{AM}}_{H^{\prime },W^{\prime }}$), ${\mathrm{AM}}_{i,j}$∈{0,1}, is expressed as

(6) $${\mathrm{AM}}_{i,j}=\left\{\begin{array}{cc} 1, & \mathrm{if}\;U_{i,j,\mathrm{ind}(a_{j})}\geq \alpha \wedge a_{j}\neq \epsilon \\ 0, & \mathrm{otherwise}, \end{array}\right.$$

where *j* is a prediction timestep or frame. $a_{j}$ is a CTC predicted character at *j*. $U_{i,j,\mathrm{ind}(a_{j})}$ is a probability of character, $a_{j}$, at timestep, *j*, and height, *i*. ind() is a character-to-index mapping. α is a threshold between zero and one while ϵ is a blank token required by a CTC decoder. A high α associates a predicted character, $a_{j}$, with the high probability image regions. The resulting character regions are illustrated in Figure 1(3).

#### 2.1.4. Alignment Evaluation Metric (AEM)

Model predictions are explicable through visualization of the association and cross-attention maps (Figure 1). We also quantitatively assess alignment between character positions in these maps and the ground truth character locations. Given the absence of explicit character coordinate predictions by the association and cross-attention maps, the intersection-over-union (IoU) metric is unsuitable. Instead, we introduce an alignment metric suitable for both association and cross-attention maps.

**Figure 4:**
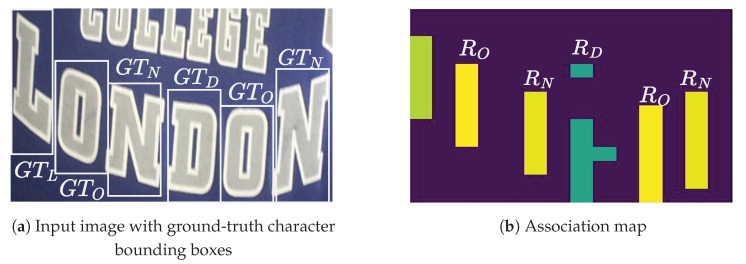
The estimated character locations, $R_{k}$, from the association map. (**a**) Input image with ground-truth character bounding boxes, ${GT}_{k}$. (**b**) Estimated character regions. Best viewed in color.

Concretely, given character regions $R_{k}$ on the association map and ${\mathrm{GT}}_{k}$ as the ground-truth bounding box (depicted in Figure 4), the alignment evaluation metric (AEM) for a predicted character, *k*, is given by

(7) $${\mathrm{AEM}}_{k}=\left\{\begin{array}{cc} 1, & \mathrm{if}\;R_{k}\cap {\mathrm{GT}}_{k}\neq 0\\ 0, & \mathrm{otherwise}. \end{array}\right.$$

The AEM for a given text of length, *L*, is given by

(8) $${\mathrm{AEM}}_{TEXT}=\frac{\sum_{k=1}^{L}{\mathrm{AEM}}_{k}}{L}.$$

**Figure 5:**
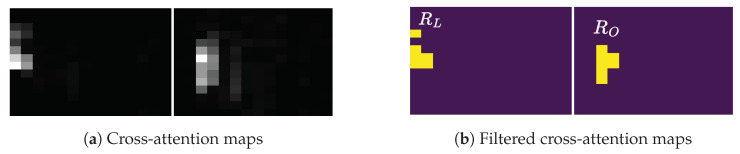
The estimated character locations, $R_{k}$, for the two predicted characters of the input image in Figure 4a, from the cross-attention maps. (**a**) Cross-attention maps. (**b**) Estimated character regions. Best viewed in color.

In the case of the cross-attention map, we first sum the cross-attention map over all attention heads in the case of multi-headed attention mechanism and normalize for each predicted character, *k*, to obtain $\mathrm{CA}=({\mathrm{CA}}_{1,1},\ldots ,{\mathrm{CA}}_{H^{\prime },W^{\prime }}$), ${\mathrm{CA}}_{i,j}$∈$R|0\leq {\mathrm{CA}}_{i,j}\leq 1$. Examples of the resulting overlaid and normalized cross-attention map are given in Figure 5a. In contrast to the association map, the cross-attention map is more diffuse due to the decoder’s need to compute continuous attention weights across the entire feature maps. We filter out regions with low attention weights below the threshold, β. The filtered, binary cross-attention map in Figure 5b, $\mathrm{CAF}=({\mathrm{CAF}}_{1,1},\ldots ,{\mathrm{CAF}}_{H^{\prime },W^{\prime }}$), ${\mathrm{CAF}}_{i,j}$∈{0,1}, is given by

(9) $${\mathrm{CAF}}_{i,j}=\left\{\begin{array}{cc} 1, & \mathrm{if}\;{\mathrm{CA}}_{i,j}\geq \beta \\ 0, & \mathrm{otherwise}. \end{array}\right.$$

where β is between zero and one. A high β associates a predicted character, *k*, with the high attention weight regions. With the CAF, ${\mathrm{AEM}}_{k}$ and ${\mathrm{AEM}}_{TEXT}$ are computed, according to the above equations.

### 2.2. Datasets

#### 2.2.1. Synthetic Datasets

**Figure 6:**
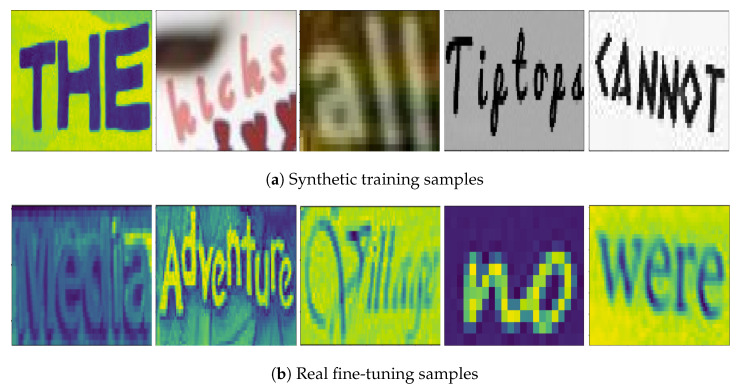
Sample training and fine-tuning images. (**a**) Sample images from the training datasets. (**b**) Sample images from the fine-tuning datasets.

Training on large-scale synthetic data is a common practice in STR. Four major synthetic datasets are MJSynth (MJ) [36], SynthText (ST) [37], SynthAdd (SA) [9], and SynthTiger [38]. The synthetic training set comprises 8.5M images from 50% of MJSynth, 50% of SynthText, 100% of SynthAdd, and 10% of SynthTiger. The mixing ratio is around 4:3:1.3:1. Combining different training sources is to increase diversity of training data. Some samples from the training datasets are shown in Figure 6a.

#### 2.2.2. Real Datasets

The evaluation datasets include the test sets of street view text (SVT) [39], IIIT5k-Words (IIIT) [40], ICDAR2013 (IC13) [41], ICDAR2015 (IC15) [42], SVT perspective (SVTP) [43], and CUTE80 (CT) [44]. Detailed descriptions of these datasets can be referred to [14,45].

COCO-Text (COCO) [46], RCTW [47], Uber-Text [48], ArT [49], LSVT [50], ReCTS [51], TextOCR [52], and OpenImages V5 [24] are small-scale, real labeled datasets. We used an aggregated, processed version of COCO-Text, RCTW, Uber-Text, ArT, LSVT, and ReCTS provided by Baek et al. [45]. For TextOCR and OpenImages V5, we used the processed versions provided by Yang et al. [5].

The fine-tuning datasets comprise the training sets of SVT, IIIT, IC03, IC13, IC15, and the real labeled datasets above. The idea of introducing the fine-tuning datasets based on real labeled data is to identify whether our ViT-CTC models have any inherent weaknesses or if there are any blindspots in the training datasets [53]. The fine-tuning datasets comprise 2.4M labeled images. A few samples from the fine-tuning datasets are shown in Figure 6b.

#### 2.2.3. Synthetic Character-Level Annotation Dataset

**Figure 7:**
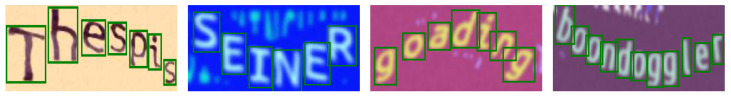
Sample text images with character-level annotations.

Character-level annotations are not available with the existing datasets. Thus, to quantitatively evaluate the character locations derived from the association and cross-attention maps, we use SynthTiger (https://github.com/clovaai/synthtiger, accessed on 1 August 2023) to synthetically generate a small dataset of 446 scene text images with character-level bounding boxes. A few samples of the generated images with character-level annotations are given in Figure 7.

### 2.3. Experiment Design

We experimented with different backbones, including three variants of DeiT-III [29] (DeiT-Small, DeiT-Medium, and DeiT-Base) and a CaiT-Small [30]. The assessment of the backbone’s complexity impact on recognition performance can be achieved by employing the DeiT-Small, DeiT-Medium, and DeiT-Base backbones. Furthermore, the inclusion of CaiT-Small enables us to compare the recognition performance of different ViT architectures.

The details of these four pretrained ViT backbones are shown in Table 1. For an input image of 224 × 224 pixels, the output feature maps are 14 × 14 × *D*, and *D* is the embedding dimension, which is provided in the same table for each ViT backbone. For each pretrained ViT backbone, we setup two ViT-CTC models, employing both the baseline feature averaging method (FA) [5] and the proposed marginalization method (M), presented in Section 2.1.1. In FA, feature maps are arithmetically averaged along the height or vertical dimension to produce a 1D feature sequence for a character classifier and a CTC decoder. As a result, it does not provide character location information.

Similarly, for recognition performance and latency comparison purposes, we also setup the Transformer-decoder-based models that are also based on the same ViT backbones, while the specifications of the Transformer decoder are provided in Table 2. It should be noted that only our ViT-CTC models using the proposed marginalization method and the Transformer-decoder-based models can offer character locations in addition to recognition. The estimated character locations are qualitatively and quantitatively evaluated against the ground-truth locations.

In the case of a CTC decoder, the character set comprises 37 characters, encompassing case-insensitive letters, numbers, and a blank token denoted as ϵ. On the other hand, for a Transformer decoder, the character set consists of 39 characters, including case-insensitive letters, numbers, and three distinct special tokens (PADDING: zero padding; EOS: end of sentence; SOS: start-of-sentence). The input images were resized to 224 × 224 pixels.

The training strategy comprised two phases: (1) training on the synthetic datasets and (2) fine-tuning on the real datasets. These two phases of training allow us to identify models’ weaknesses or training datasets’ blindspots during evaluation [53]. The training process lasted for 50 iterations. During each iteration, 300,000 images were randomly selected, and a batch of 64 images was used for training without any data augmentation to ensure a fair comparison with the SOTA methods [4,14]. In addition, because the synthetically generated training images were already augmented during generation, additional data augmentation, such as [54,55], may affect the recognition accuracy negatively [45]. The total training is equivalent to around two epochs on all of the training data. The fine-tuning phase followed the same settings as before, but it only lasted for 30 iterations, which is approximately equivalent to three epochs over the entire fine-tuning dataset. The cyclic learning schedules between $10^{-4}$ and $10^{-5}$ and between $10^{-5}$ and $10^{-6}$ were used for the training and fine-tuning phases, respectively. For all the models, pretrained ViT weights [29,30] were used with a gradient clip of ten.

## 3. Results

In this section, we present the experimental outcomes and important analyses. To evaluate the performance of our ViT-CTC models using the proposed method (M), we begin by providing the ablation analyses of the encoder complexities and architectures in Section 3.1, followed by comparing their accuracy with the baseline and SOTA-based methods that do not provide character locations in Section 3.2 and Section 3.3. In Section 3.4, we compare with the baseline Transformer-decoder-based models that provide character locations via the cross-attention map. Lastly, we provide the qualitative and quantitative evaluation of character location derived from the proposed method and the cross-attention map.

### 3.1. Ablation Analyses of the Encoder Complexities and Architectures

In this section, we present the ablation analyses concerning ViT-based feature extractor complexities since the feature extractor is the main component in the proposed method. We utilize three variants of DeiT backbones (namely DeiT-S, DeiT-M, and DeiT-B) and explore different encoder architectures employing a CaiT-S backbone.

Table 3 demonstrates that increasing the complexity of the ViT-based feature extractor, specifically transitioning from DeiT-S to DeiT-M and DeiT-B, results in higher total word recognition accuracy for both synthetic and real training data. However, these improvements are accompanied by larger model sizes and heightened computational demands, as indicated in Table 1. Table 3 also shows that despite having a much smaller model size and computational demand, the CaiT-S model achieves a comparable total recognition accuracy with the DeiT-B model for both synthetic and real training data.

### 3.2. Recognition Accuracy Comparison with the Baseline Feature Averaging

In this section, we perform a comparison to assess the recognition accuracy of our ViT-CTC models using both the proposed method (M) and the baseline feature averaging (FA). Since FA does not yield character localization, the comparison in this section primarily centers around the recognition accuracy between the two methods.

As indicated in Table 4a,b, there are minimal distinctions in terms of recognition accuracy between the two methods, regardless of source of training data (i.e., real and synthetic). The findings can be distilled into three primary points. Firstly, the proposed method, offering both model explainability and character location information, does not lead to any loss of recognition accuracy. Secondly, the utilization of a 2D feature extractor such as a ViT backbone improves the recognition accuracy of a CTC decoder, whereas the majority of CTC-based methods depend on a tailored 1D feature extractor. Thirdly, the utilization of real labeled data, albeit limited, results in a substantial recognition performance improvement compared with relying solely on synthetic training data.

### 3.3. Recognition Accuracy Comparison with the SOTA CTC-Based Methods

Similar to the preceding section, this section compares the recognition accuracy of our ViT-CTC models using our proposed method (M) with the SOTA CTC-based methods lacking character location information. Among the SOTA methods in Table 5, only the DiG-ViT [5] and GTC [4] models use real labeled data for training. The other models use solely synthetic data for training. The table suggests that integrating real labeled data can improve recognition accuracy on benchmark datasets. However, various factors like backbone architecture, training iterations, and data augmentation also play a significant role in this improvement. Among these methods, only ViTSTR [1] and DiG-ViT employ a ViT backbone; the rest rely on convolutional backbones. DiG-ViT employs the feature averaging technique to convert 2D feature maps to 1D for a CTC decoder. GTC [4] uses an attention-based decoder to guide a CTC decoder.

Focusing on the models trained only on synthetic data (S), Table 5a shows that our ViT-CTC models using the proposed method (M) outperform the SOTA CTC-based methods, such as TRBC (TPS-ResNet-BiLSTM-CTC) [14], in recognition accuracy (bold numbers in the table). This recognition accuracy improvement is attributed to the advanced feature extraction of pretrained ViT backbones. Meanwhile, when considering methods trained or fine-tuned on real labeled data (R), Table 5b shows that our ViT-CTC models slightly outperform the SOTA DiG-ViT models (bold numbers in the table). Thus, regardless of the training data source, our ViT-CTC models with the proposed method (M) consistently show superior or comparable performance to the SOTA CTC-based methods.

### 3.4. Recognition Accuracy and Efficiency Comparison with the Baseline Transformer-Decoder-Based Models

Earlier sections evaluated our proposed ViT-CTC models’ recognition accuracy against the CTC-based methods that lack character localization. Now, we jointly compare recognition accuracy and latency with a Transformer-decoder-based architecture that can associate predicted characters with relevant image regions.

A CTC decoder is acknowledged for its faster inference but lower recognition accuracy compared with a Transformer decoder that learns an implicit language model [3,4,5,14]. This section quantitatively assesses the trade-off between the two decoders in terms of both latency and recognition accuracy.

Table 6a,b compare the recognition accuracy of our ViT-CTC models using our proposed method against Transformer-decoder-based models. Regardless of the training data source, the Transformer-decoder-based models consistently achieved higher recognition accuracy on benchmark datasets due to their ability to capture character dependencies through implicit language modeling that is absent in a CTC decocder.

**Figure 8:**
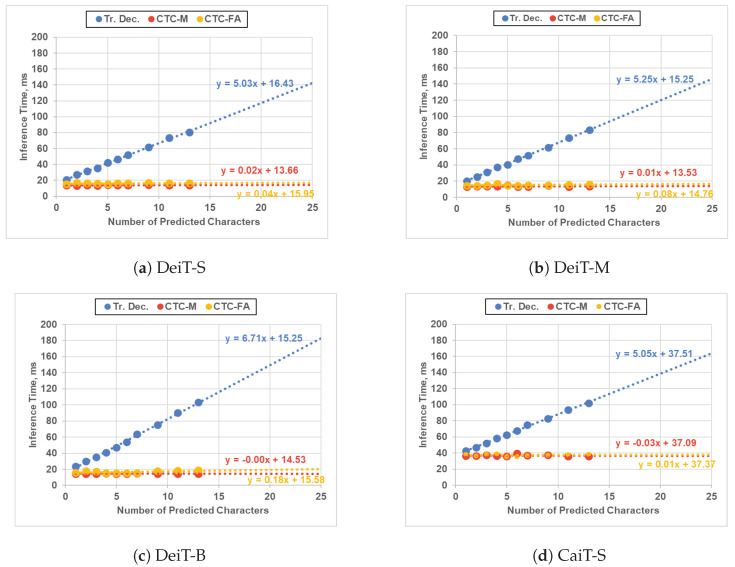
Inference time comparison between our ViT-CTC models and the Transformer-decoder-based models on an RTX 2060 GPU. Trendlines are projected to the maximum number of characters (i.e., 25) [1]. Tr. Dec.: Transformer decoder. CTC-M: CTC decoder with the proposed method. CTC-FA: CTC decoder with feature averaging. Best viewed in color.

However, this recognition accuracy advantage was offset by increased latency, as shown in Figure 8 and Table 7. The inference time of a Transformer decoder is directly tied to the number of decoded characters, while a CTC decoder maintains a constant inference time. Quantitatively, the inference speed of a CTC decoder surpasses a Transformer decoder by up to 12 times, making it more appealing in low-latency and low-resource scenarios.

**Figure 9:**
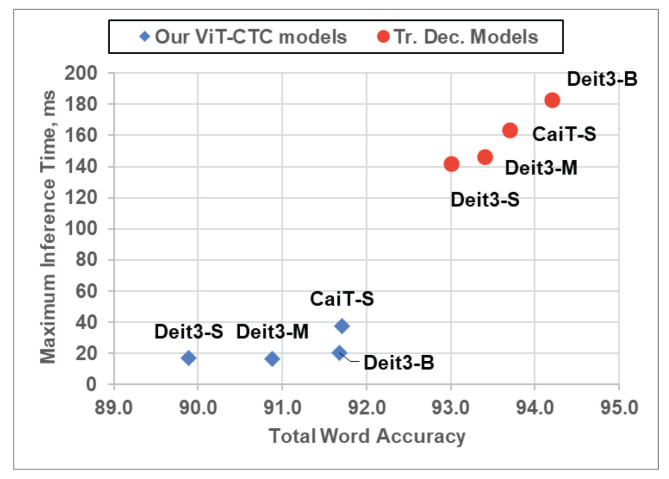
Maximum inference time vs. recognition accuracy comparisons between the ViT-CTC models using the proposed method and the Transformer-decoder-based models on an RTX 2060 GPU. Tr. Dec.: Transformer decoder. Best viewed in color.

Considering both latency and recognition accuracy, Figure 9 summarizes the trade-off between a CTC decoder and a Transformer decoder using different ViT backbones. With the same ViT backbone, the CTC decoder outperforms the Transformer decoder significantly in terms of efficiency, with a speed advantage of up to 12 times. However, this speed gain is countered by a maximum reduction in overall word recognition accuracy of 3.1%.

### 3.5. Qualitative Evaluation of Association Map

Until now, we have examined our ViT-CTC models’ recognition performance and efficiency in comparison to the CTC and Transformer-decoder-based models. This section shifts focus to the significance of the association map, denoted as AM, which is a key output of our proposed method. The detailed derivation of the AM can be found in Section 2.1.3. Utilizing an AM enables the establishment of explainable 2D spatial relationships between the model’s predictions and relevant image regions. This spatial link is crucial for understanding the model’s predictions and localization. The AM generated by our proposed method corresponds to the cross-attention map formed by the cross-attention module within the Transformer decoder. This module selectively incorporates relevant features for adaptive character predictions.

**Figure 10:**
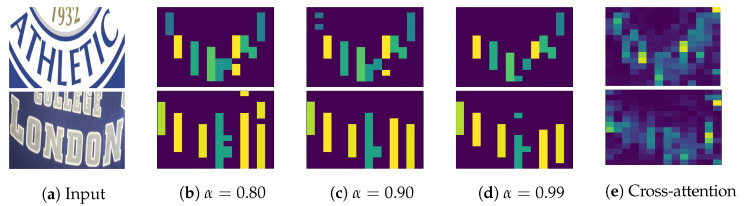
Association maps for different values of α. The color bars show image regions, corresponding to predicted characters. Best viewed in color.

Figure 10 displays the association maps corresponding to different α values for two examples where text from the top intrudes. Instead of ‘1932’ and ‘COLLEGE’, the ground-truth words are ‘ATHLETIC’ and ‘LONDON’. The ViT-CTC model accurately predicts both words. Examination of the association maps reveals the model accurately linking the correctly predicted characters with the relevant lower regions containing ‘ATHLETIC’ and ‘LONDON’, as opposed to upper regions with ‘1932’ and ‘COLLEGE’. Thus, association maps not only explain the model’s predictions but also offer localization for those predictions.

As α increases, the association maps maintain high probability regions while discarding those below α, as seen in Figure 10d. Compared with the Transformer decoder’s cross-attention maps in Figure 10e, overall alignments are observed. These alignments validate the accuracy and reliability of the association maps from our proposed method that does not reply on a computationally-intensive cross-attention mechanism.

### 3.6. Quantitative Evaluation of Association Maps

In this section, we quantitatively evaluate our ViT-CTC models’ association map and the Transformer-decoder-based models’ cross-attention map. Employing Equation (6) for the association map and Equation (9) for the cross-attention map, we calculate alignment evaluation metrics (AEMs) using Equation (8). This was performed using different threshold values α and β, respectively, on the synthetic dataset with character-level annotations, as detailed in Section 2.2.3. To ensure fairness, only image samples correctly recognized by both our ViT-CTC and the Transformer-decoder-based models were included in the evaluation.

**Figure 11:**
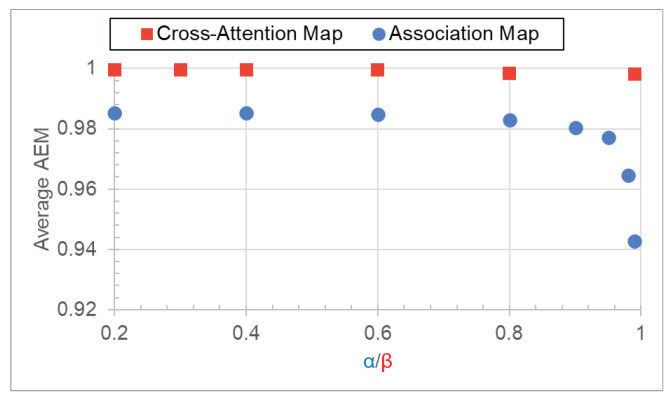
The average AEMs of the association and cross-attention maps as a function of α and β, respectively. Best viewed in color.

Figure 11 depicts that the average alignment evaluation metric (AEM) of the cross-attention map remains stable across different β values, showing good alignment accuracy with the ground-truth character locations. In contrast, the average AEM of the association map exhibits slight sensitivity to α, particularly at higher values. For α≤0.95, the average AEM of the association map remains above 98% accuracy, signifying strong alignment between the estimated and ground-truth character locations. Thus, the association map is comparable to the cross-attention map in localizing the predicted characters, while the former has a significantly lower computational demand.

**Figure 12:**
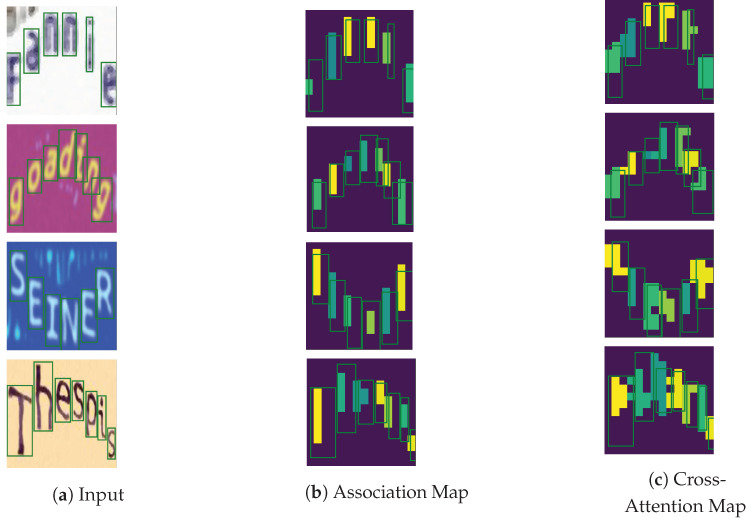
Illustrations of the estimated character locations from the association (α=0.8) and cross-attention (β=0.5) maps vs. the ground-truth character locations. Best viewed in color.

Figure 12 compares the estimated character locations from the association and cross-attention maps with the ground-truth bounding boxes in a few highly curved text images. Both methods’ estimated character locations closely align with the ground-truth positions.

## 4. Limitations and Future Work

Since a CTC decoder is many to one, the pretrained ViT backbone must produce 2D feature maps, the width of which must be greater than or equal to the length of text in an input image. For a ViT-CTC backbone that takes an input image of 224 × 224 pixels and returns 14 × 14 feature maps, it can predict at most 14 characters. Moreover, due to its reliance on left-to-right alignments, a CTC decoder is unable to recognize vertical or highly oriented text images.

Furthermore, due to the sizable receptive field of 16 × 16 pixels in the pretrained ViT backbones employed in this research, the character locations they generate exhibit low resolution.

Thus, future experiments will consider other pretrained ViT or hybrid CNN-Transformer backbones that output dense feature maps, increasing the number of predicted characters and enhancing the resolution of the resulting association map. We will also explore two potential applications of the association maps. Firstly, the association map can guide a Transformer decoder to counter attention drift in long textline images. Secondly, estimated character locations can aid text rectification for highly curved text images.

## 5. Conclusions

In this paper, we propose a marginalization-based method that enables a 2D feature extractor with a 1D CTC decoder by predicting an output sequence of 2D joint probability distributions over the height and class dimensions. The height dimension is marginalized to suit a 1D CTC decoder. In addition, the proposed method yields an association map that can be used to determine character locations and explain model predictions.

The experimental results show that our ViT-CTC models outperform the recent CTC-based SOTA methods on the public benchmark datasets in terms of recognition accuracy. Compared with a Transformer-decoder-based model, a ViT-CTC model has a maximum reduction in total word recognition accuracy of 3.1%, regardless of the ViT backbone. However, a ViT-CTC model exhibits a substantial speed improvement, surpassing a Transformer-decoder-based model by up to 12 times. Both the qualitative and quantitative evaluations of the character locations estimated from the association map closely correspond with those estimated using the cross-attention map and the ground-truth character-level bounding boxes.

## Figures and Tables

**Table 1:** Specifications of the pretrained ViT backbones.

ViT Model	Params	GFLOPs	Size	Emb. Dim, *D*	Acc@1 (INet-1k)
DeiT-Small [30]	22.2 M	4.6	224 × 224	384	81.4
DeiT-Medium [30]	38.8 M	8.0	224 × 224	512	83.0
DeiT-Base [30]	86.6 M	17.5	224 × 224	768	83.8
CaiT-Small [29]	47.0 M	9.4	224 × 224	384	83.5

**Table 2:** Specifications of the Transformer decoder.

Parameters	Value
Hidden/Embedding Dimension	Encoder’s Emb. Dim.
Decoder Stacks	3
Attention Heads	8
Dropout	0.1
Feed-forward Dimension	Encoder’s Emb. Dim.

**Table 3:** Word recognition accuracy (%) of the ablation results of the encoder complexities and architectures with the proposed method (M). FT: fine-tuning on real data. Bold: highest.

(a) Methods Trained on Synthetic Training Data (S).							
**Method**	**IIIT**	**SVT**	**IC13**	**IC15**	**SVTP**	**CUTE**	**Total**
**DeiT-S + M (Ours)**	91.4	85.5	91.3	75.3	76.7	82.2	85.3
**DeiT-M + M (Ours)**	92.5	**87.8**	**92.2**	76.6	**79.5**	81.9	86.6
**DeiT-B + M (Ours)**	93.0	86.9	92.2	**78.6**	79.1	84.0	**87.3**
**CaiT-S + M (Ours)**	**93.5**	86.9	91.9	77.6	77.8	**85.4**	87.2
**(b) Methods Trained on Real Labeled Training Data (R).**							
**Method**	**IIIT**	**SVT**	**IC13**	**IC15**	**SVTP**	**CUTE**	**Total**
**DeiT-S + M + FT (Ours)**	94.6	89.2	95.4	81.5	83.1	91.3	89.9
**DeiT-M + M + FT (Ours)**	95.0	92.3	95.2	83.5	84.0	90.9	90.9
**DeiT-B + M + FT (Ours)**	95.9	**92.6**	**96.1**	84.4	84.3	**92.7**	**91.7**
**CaiT-S + M + FT (Ours)**	**96.1**	90.6	95.4	**84.9**	**85.4**	**92.7**	**91.7**

**Table 4:** Word recognition accuracy (%) comparison between the proposed method (M) and the baseline feature averaging (FA). FT: fine-tuning on real data. Bold: highest.

(a) Methods Trained on Synthetic Training Data (S).							
**Method**	**IIIT**	**SVT**	**IC13**	**IC15**	**SVTP**	**CUTE**	**Total**
**DeiT-S + FA**	91.4	86.4	89.6	74.2	75.8	79.1	84.7
**DeiT-M + FA**	92.0	87.3	91.4	77.4	78.9	82.2	86.4
**DeiT-B + FA**	93.1	**88.7**	**92.9**	77.3	**79.7**	85.7	87.4
**CaiT-S + FA**	**94.3**	87.2	92.5	**79.5**	79.4	**87.1**	**88.2**
**DeiT-S + M (Ours)**	91.4	85.5	91.3	75.3	76.7	82.2	85.3
**DeiT-M + M (Ours)**	92.5	87.8	92.2	76.6	79.5	81.9	86.6
**DeiT-B + M (Ours)**	93.0	86.9	92.2	78.6	79.1	84.0	87.3
**CaiT-S + M (Ours)**	93.5	86.9	91.9	77.6	77.8	85.4	87.2
**(b) Methods Trained on Real Labeled Training Data (R).**							
**Method**	**IIIT**	**SVT**	**IC13**	**IC15**	**SVTP**	**CUTE**	**Total**
**DeiT-S + FA + FT**	95.0	88.4	94.2	81.6	82.0	88.5	89.6
**DeiT-M + FA + FT**	95.5	91.2	95.4	83.4	83.4	92.0	91.0
**DeiT-B + FA + FT**	95.9	92.1	95.9	83.9	84.2	92.7	91.5
**CaiT-S + FA + FT**	96.0	92.3	95.8	84.5	84.7	**93.7**	**91.7**
**DeiT-S + M + FT (Ours)**	94.6	89.2	95.4	81.5	83.1	91.3	89.9
**DeiT-M + M + FT (Ours)**	95.0	92.3	95.2	83.5	84.0	90.9	90.9
**DeiT-B + M + FT (Ours)**	95.9	**92.6**	**96.1**	84.4	84.3	92.7	**91.7**
**CaiT-S + M + FT (Ours)**	**96.1**	90.6	95.4	**84.9**	**85.4**	92.7	**91.7**

**Table 5:** Word recognition accuracy (%) comparison between the proposed method (M) and the SOTA CTC-based methods. FT: fine-tuning on real data. Size: parameters in millions. M: the proposed method. Bold: highest.

(a) Methods Trained on Synthetic Training Data (S).								
**Method**	**Size**	**IIIT**	**SVT**	**IC13**	**IC15**	**SVTP**	**CUTE**	**Total**
CRNN [15]	8.3	82.9	81.6	89.2	69.4	70.0	65.5	78.5
STAR-Net [18]	48.7	87.0	86.9	91.5	76.1	77.5	71.7	83.5
GRCNN [17]	4.6	84.2	83.7	88.8	71.4	73.6	68.1	80.1
Rosetta [16]	44.3	84.3	84.7	89.0	71.2	73.8	69.2	80.3
TRBC [14]	48.7	87.0	86.9	91.5	76.1	77.5	71.7	83.5
ViTSTR-S [1]	21.5	85.6	85.3	90.6	75.3	78.1	71.3	82.5
ViTSTR-B [1]	85.8	86.9	87.2	91.3	76.8	**80.0**	74.7	84.0
**DeiT-S + M (Ours)**	21.6	91.4	85.5	91.3	75.3	76.7	82.2	85.3
**DeiT-M + M (Ours)**	38.9	92.5	**87.8**	**92.2**	76.6	79.5	81.9	86.6
**DeiT-B + M (Ours)**	**85.7**	93.0	86.9	**92.2**	**78.6**	79.1	84.0	**87.3**
**CaiT-S + M (Ours)**	46.5	**93.5**	86.9	91.9	77.6	77.8	**85.4**	87.2
**(b) Methods Trained on Real Labeled Training Data (R).**								
**Method**	**Size**	**IIIT**	**SVT**	**IC13**	**IC15**	**SVTP**	**CUTE**	**Total**
GTC [4]	-	96.0	91.8	93.2	79.5	85.6	91.3	90.1
DiG-ViT-T (CTC) [5]	20.0	93.3	89.7	92.5	79.1	78.8	83.0	87.7
DiG-ViT-S (CTC) [5]	36.0	95.5	91.8	95.0	84.1	83.9	86.5	91.0
DiG-ViT-B (CTC) [5]	52.0	95.9	92.6	95.3	84.2	85.0	89.2	91.5
**DeiT-S + M + FT (Ours)**	21.6	94.6	89.2	95.4	81.5	83.1	91.3	89.9
**DeiT-M + M + FT (Ours)**	38.9	95.0	92.3	95.2	83.5	84.0	90.9	90.9
**DeiT-B + M + FT (Ours)**	**85.7**	95.9	**92.6**	**96.1**	84.4	84.3	**92.7**	**91.7**
**CaiT-S + M + FT (Ours)**	46.5	**96.1**	90.6	95.4	**84.9**	**85.4**	**92.7**	**91.7**

**Table 6:** Word recognition accuracy (%) comparison with the baseline Transformer-decoder-based models. FT: fine-tuning on real data. Size: parameters in millions. Tr. Dec.: Transformer decoder. M: the proposed method. Bold: highest.

(a) Methods Trained on Synthetic Training Data (S).								
**Method**	**Size**	**IIIT**	**SVT**	**IC13**	**IC15**	**SVTP**	**CUTE**	**Total**
**DeiT-S + Tr. Dec.**	26.1	93.7	88.9	92.4	80.0	80.6	86.8	88.3
**DeiT-M + Tr. Dec.**	46.2	94.1	89.6	92.6	**81.5**	82.8	83.6	89.0
**DeiT-B + Tr. Dec.**	**103.4**	94.8	**90.3**	92.9	81.0	**85.1**	87.5	89.6
**CaiT-S + Tr. Dec.**	50.9	**94.9**	**90.3**	**94.2**	81.3	83.4	**89.9**	**89.9**
**DeiT-S + M (Ours)**	21.6	91.4	85.5	91.3	75.3	76.7	82.2	85.3
**DeiT-M + M (Ours)**	38.9	92.5	87.8	92.2	76.6	79.5	81.9	86.6
**DeiT-B + M (Ours)**	85.7	93.0	86.9	92.2	78.6	79.1	84.0	87.3
**CaiT-S + M (Ours)**	46.5	93.5	86.9	91.9	77.6	77.8	85.4	87.2
**(b) Methods Trained on Real Labeled Training Data (R).**								
**Method**	**Size**	**IIIT**	**SVT**	**IC13**	**IC15**	**SVTP**	**CUTE**	**Total**
**DeiT-S + Tr. Dec. + FT**	26.1	96.8	93.0	96.7	86.3	87.8	94.8	93.0
**DeiT-M + Tr. Dec. + FT**	46.2	97.0	94.0	97.1	86.3	89.3	95.1	93.4
**DeiT-B + Tr. Dec. + FT**	**103.4**	**98.0**	94.6	**97.5**	**86.9**	**90.5**	95.1	**94.2**
**CaiT-S + Tr. Dec. + FT**	50.9	97.4	**94.9**	97.1	86.5	89.5	**95.8**	93.7
**DeiT-S + M + FT (Ours)**	21.6	94.6	89.2	95.4	81.5	83.1	91.3	89.9
**DeiT-M + M + FT (Ours)**	38.9	95.0	92.3	95.2	83.5	84.0	90.9	90.9
**DeiT-B + M + FT (Ours)**	85.7	95.9	92.6	96.1	84.4	84.3	92.7	91.7
**CaiT-S + M + FT (Ours)**	46.5	96.1	90.6	95.4	84.9	85.4	92.7	91.7

**Table 7:** Maximum inference time comparison. Bold: highest. FA: feature averaging. M: the proposed method. Tr. Dec.: Transformer decoder.

Method	GFLOPs	Time (ms)
**DeiT-S + Tr. Dec.**	4.9	142
**DeiT-M + Tr. Dec.**	8.5	146
**DeiT-B + Tr. Dec.**	**18.7**	**183**
**CaiT-S + Tr. Dec.**	9.6	164
**DeiT-S + FA**	4.6	17
**DeiT-M + FA**	8.0	17
**DeiT-B + FA**	17.5	20
**CaiT-S + FA**	9.4	38
**DeiT-S + M (Ours)**	4.6	14
**DeiT-M + M (Ours)**	8.0	14
**DeiT-B + M (Ours)**	17.5	15
**CaiT-S + M (Ours)**	9.4	36

## Data Availability

Available upon request.
